# Characterization of Phage Resistance and Their Impacts on Bacterial Fitness in Pseudomonas aeruginosa

**DOI:** 10.1128/spectrum.02072-22

**Published:** 2022-09-21

**Authors:** Na Li, Yigang Zeng, Mengran Wang, Rong Bao, Yu Chen, Xiaoyu Li, Jue Pan, Tongyu Zhu, Bijie Hu, Demeng Tan

**Affiliations:** a Department of Infectious Diseases, Zhongshan Hospital, Fudan University, Shanghai, China; b Shanghai Public Health Clinical Center, Fudan University, Shanghai, China; c Department of Laboratory Medicine, Zhongshan Hospital, Fudan University, Shanghai, China; d School of Bioengineering, Dalian University of Technology, Dalian, China; Institut Pasteur

**Keywords:** phage resistance, phage-host interactions, *Pseudomonas aeruginosa*, phage therapy

## Abstract

The emergence and spread of antibiotic resistance pose serious environmental and health challenges. Attention has been drawn to phage therapy as an alternative approach to combat antibiotic resistance with immense potential. However, one of the obstacles to phage therapy is phage resistance, and it can be acquired through genetic mutations, followed by consequences of phenotypic variations. Therefore, understanding the mechanisms underlying phage-host interactions will provide us with greater detail on how to optimize phage therapy. In this study, three lytic phages (phipa2, phipa4, and phipa10) were isolated to investigate phage resistance and the potential fitness trade-offs in Pseudomonas aeruginosa. Specifically, in phage-resistant mutants phipa2-R and phipa4-R, mutations in conferring resistance occurred in genes *pilT* and *pilB,* both essential for type IV pili (T4P) biosynthesis. In the phage-resistant mutant phipa10-R, a large chromosomal deletion of ~294 kb, including the *hmgA* (homogentisate 1,2-dioxygenase) and *galU* (UTP–glucose-1-phosphate uridylyltransferase) genes, was observed and conferred phage phipa10 resistance. Further, we show examples of associated trade-offs in these phage-resistant mutations, e.g., impaired motility, reduced biofilm formation, and increased antibiotic susceptibility. Collectively, our study sheds light on resistance-mediated genetic mutations and their pleiotropic phenotypes, further emphasizing the impressive complexity and diversity of phage-host interactions and the challenges they pose when controlling bacterial diseases in this important pathogen.

**IMPORTANCE** Battling phage resistance is one of the main challenges faced by phage therapy. To overcome this challenge, detailed information about the mechanisms of phage-host interactions is required to understand the bacterial evolutionary processes. In this study, we identified mutations in key steps of type IV pili (T4P) and O-antigen biosynthesis leading to phage resistance and provided new evidence on how phage predation contributed toward host phenotypes and fitness variations. Together, our results add further fundamental knowledge on phage-host interactions and how they regulate different aspects of Pseudomonas cell behaviors.

## INTRODUCTION

Pseudomonas aeruginosa is a Gram-negative opportunistic pathogen which can cause severe respiratory infections, urinary tract infections, and blood infections in immunocompromised patients (1). The overuse and misuse of antibiotics select for multidrug-resistant (MDR) bacterial pathogens and further endanger the efficacy and development of antibiotics. The tolerance and persistence of P. aeruginosa in nosocomial infections is due to its ability to produce exopolysaccharide capsules, forming biofilms with increased resistance to antibiotics and phagocytosis (2, 3). Therefore, the development of alternative therapeutic and prophylactic approaches to traditional antibiotics for controlling and preventing P. aeruginosa infection is needed.

Bacteriophages, or “phages,” are one the most abundant and diverse entities on earth (4). Phages exhibit two distinct life cycles that can be classified into lytic or lysogenic depending on their interactions with host cells. In the lytic cycle, the infecting phages ultimately kill their host cells and subsequently release new phage progenies. In contrast, the lysogenic cycle is where phages integrate their genomes into their hosts and replicate along with the bacterial life cycle, remaining in a quiescent state without killing the host (4). Conventionally, lytic phages have preferably been chosen and harnessed to treat pathogenic bacterial infections (5). Recent studies have explored phage treatments against a range of clinical pathogens, specifically ESKAPE pathogens (Enterococcus faecium, Staphylococcus aureus, Klebsiella pneumoniae, Acinetobacter baumannii, Pseudomonas aeruginosa, and Enterobacter spp.) (6–11). While these studies have provided strong evidence toward phage therapy as a potential alternative or supplement treatment to traditional antibiotic, phage therapy is complicated by surviving subpopulations with complex genotypes and phenotypes, i.e., a wide range of conferring resistance mechanisms to phage predation (12). Thus, the evolution of phage resistance in bacterial hosts is regarded as a significant barrier to the full capacity and implementation of phage therapy (13, 14).

A commonly used phage defense strategy is to prevent phage adsorption during the initial step of the infection, often associated with loss or altered phage receptor structures to block the recognition of phage receptor-binding proteins (14). In many instances, mutations of phage receptors often have pleiotropic effects on motility, biofilm formation, and antibiotic sensitivity, making the mutants less competitive in heterogeneous populations (15, 16). In P. aeruginosa, lipopolysaccharide (LPS) and type IV pili (T4P) are the primary receptors for many phages (17–19). However, the evolution of different phenotypes and natural genetic diversification in P. aeruginosa remain to be explored, particularly the ones shaped by reciprocal evolution between bacterial hosts and their phages (20). Therefore, it is crucial to characterize how bacteria evade phage attacks, and the potential physiological outcomes of phage resistance prior to the development of any intended phage applications.

In this study, we isolated and characterized three phages against clinical isolates of P. aeruginosa. Upon examination of lytic phage activities in planktonic cultures, rapid regrowth of phage-resistant subpopulations following the initial lysis suggested that phage resistance had developed in host populations. These presumably phage-resistant mutants were further collected and characterized through the comparative genomic analysis to identify genes associated with phage resistance and potential fitness costs. Overall, our study contributes to increased knowledge about genome plasticity and phage-resistant regulation and provides a template for evaluating phages with therapeutic potential for application as phage therapy in P. aeruginosa.

## RESULTS

### Isolation and characterization of phages.

We enriched for phages capable of infecting P. aeruginosa by incubating sewage samples together with mid-log-phase growing liquid cultures of P. aeruginosa for 6 h. Each enrichment was centrifuged, filter sterilized, treated with chloroform, and spotted onto a lawn of P. aeruginosa. Of the 10 sewage samples, three produced clear plaques, which indicated the presence of lytic phages. These three plaques, herein referred to as phage phipa2, phipa4, and phipa10, were purified and revealed the host range and lytic efficiencies against 32 bacterial isolates to discriminate all isolated phages. As shown in Table 1, there were large variabilities in both host range susceptibilities and lytic efficiencies. For example, none of the phages exhibited a broad host range, infecting only a small fraction (<21.9%) of the bacterial collection (Table 1). These results suggest that host range assay may be an efficient means to discriminate phage isolates, as we considered unidentical host range may represent different phages. Consistent with the host range assay, we noticed differences among the plaque morphologies across the three phages. Specifically, phipa2 and phipa10 formed large clear plaques on strain ZS-PA-35, while phage phipa4 formed small clear plaques on strain ZS-PA-16. Interestingly, clear plaques surrounded by translucent halos were observed in phage phipa2, indicating the production of depolymerase, an enzyme capable of degrading bacterial exopolysaccharides (21).

**TABLE 1:** Host ranges of three phages were determined by spot testing aliquots of 2 μL phage lysate on LB agar overlay plates against 32 Pseudomonas aeruginosa strains

P. aeruginosa strains	Phage host range		
	phipa2	phipa4	phipa10
ZS-PA-1	^*a*^	^*c*^	
ZS-PA-2			
ZS-PA-3			
ZS-PA-4			
ZS-PA-5			
ZS-PA-7			
ZS-PA-8			
ZS-PA-9			
ZS-PA-10			
ZS-PA-11			
ZS-PA-13			
ZS-PA-14			
ZS-PA-15			^*b*^
ZS-PA-16		*^d^	
ZS-PA-17			
ZS-PA-18			
ZS-PA-19			
ZS-PA-20			
ZS-PA-21			
ZS-PA-22			
ZS-PA-23			
ZS-PA-24			
ZS-PA-25			
ZS-PA-27			
ZS-PA-28			
ZS-PA-29			
ZS-PA-30			
ZS-PA-31			
ZS-PA-32			
ZS-PA-33			
ZS-PA-34			
ZS-PA-35	*		*

a Light gray boxes indicate turbid plaques.

b Dark gray boxes indicate clear plaques.

c White boxes indicate no inhibition.

d Asterisks (*) indicate phage host.

**FIG 1:**
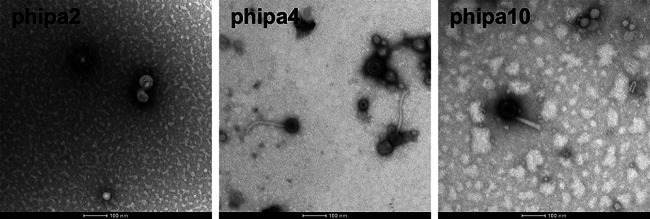
TEM micrographs of phage phipa2 (*Podoviridae*), phipa4 (*Siphoviridae*), and phipa10 (*Myoviridae*). Phage lysates were negatively stained with 2% sodium phosphotungstate. Scale bar, 100 nm.

As shown in Fig. 1, transmission electron microscopy (TEM) analysis reveals that phipa2 exhibits the typical phage morphology of the family *Podoviridae*, with an icosahedral capsid head measuring ~50 nm and short tails measuring ~17 nm long and ~13 nm wide. Phage phipa4 appears to belong to the family *Siphoviridae*, having an icosahedral capsid head (~50 nm) and a long flexible tail (~175 nm long and ~10 nm wide). The *Myoviridae* phage phipa10 carries an icosahedral head (~60 nm) and a long contractile tail (~110 nm long and ~16 nm wide). Whole-genome sequencing was performed to provide detailed analysis of the phages. Specifically, phage phipa2, phipa4, and phipa10 possess genome sizes of 43,291 bp, 42,943 bp, and 92,393 bp, respectively. Bioinformatics analysis reveals that both phipa2 and phipa4 contain 56 predicted ORFs. All ORFs of phage phipa2 are transcribed from the same strand, while the overall genome of phipa4 is transcribed into two opposite directions in which ORFs 1 to 46 are transcribed clockwise, while ORFs 47 to 56 are in the counterclockwise direction (Fig. S1). Standard nucleotide BLAST demonstrated that phipa2, phipa4, and phipa10 closely resemble that of the P. aeruginosa phage vB_Pae_QDWS (coverage 94% and identity 96.81%) (22), phage Kopi (coverage 93% and identity 96.60%) (23), and phage YS35 (coverage 96% and identity 96.76%) (24), respectively. The schematic maps of the phage genomes are listed in Fig. S1.

### Phage growth characteristics in LB broth.

**FIG 2:**
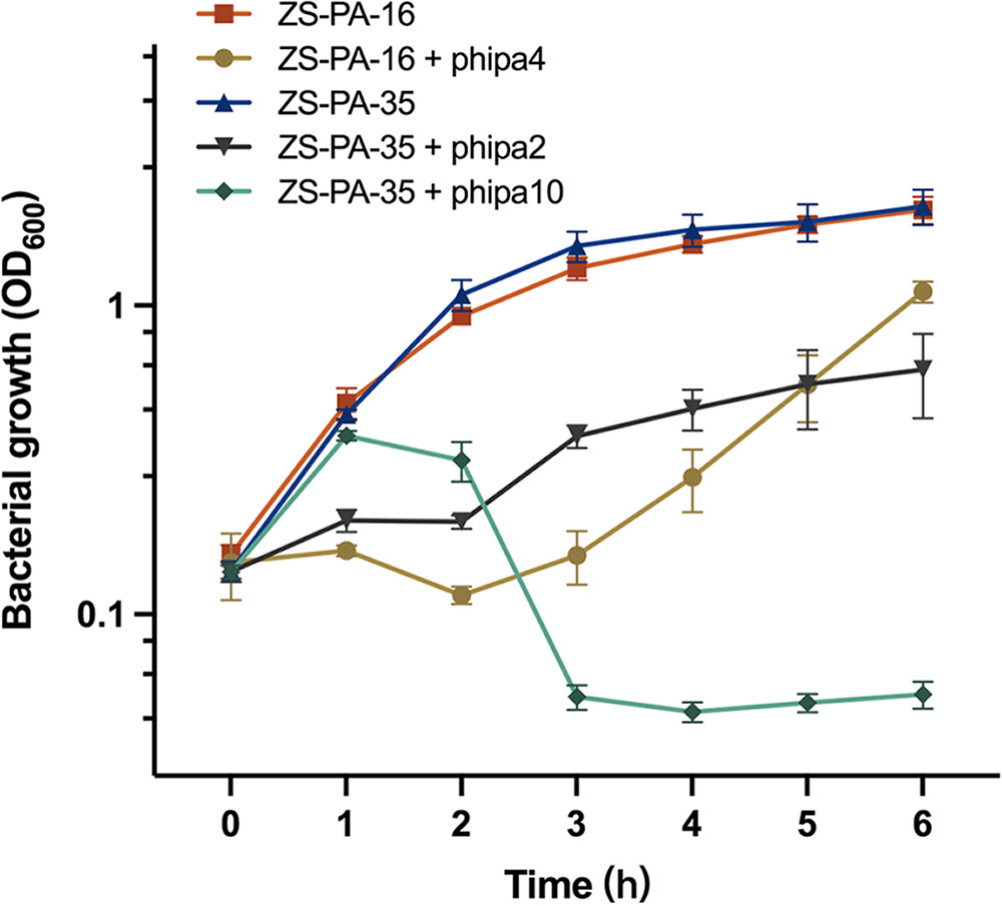
Optical density (OD600) in liquid culture for P. aeruginosa strain ZS-PA-16 in the presence or absence of phage phipa4 and strain ZS-PA-35 in the presence or absence of phage phipa2 or phipa10 (MOIs ca. 2) were measured at 1-h intervals over a 6-h period of incubation. Error bars represent standard deviations from all experiments carried out in triplicate.

To examine the efficiency of individual phipa2, phipa4, and phipa10 infections, P. aeruginosa strain ZS-PA-16 and ZS-PA-35 were challenged in liquid cultures at multiplicity of infections (MOIs) around 2. Growth kinetics were assessed by the measurement of the optical densities of the bacterial cultures with or without phage addition. As shown in Fig. 2, the growth of strain ZS-PA-16 and ZS-PA-35 without phage demonstrated a typical population growth curve with similar growth rates. In the cultures of ZS-PA-16 + phipa4, there was a drastic decrease in the growth on the initial control of the host population for 2 h, followed by exponential regrowth of the subpopulation. After 6 h, the cultures of ZS-PA-16 + phipa4 and control reached an optical density (OD600) of 1.11 and 2.04, respectively, indicating that addition of phage phipa4 had a clear impact on the growth dynamic of strain ZS-PA-16. Conversely, the addition of phage phipa10 showed totally different lytic potentials than that of strain ZS-PA-35 growth dynamics. The bacterial population density of culture ZS-PA-35 + phipa10 increased exponentially during the first 1 h, but decreased drastically in response to the predation of phage phipa10, completely reaching its plateau phase after 3 h and finally stabilizing at OD600 of ~0.05. On the other hand, the final OD in the control group of ZS-PA-35 reached 2.09, approximately 41 times higher than that of the phage phipa10-added cultures. However, the addition of phage phipa2 was less effective against the host in the cultures of ZS-PA-35 + phipa2, as suppression was only observed for ~2 h, after which growth resumed. A similar inhibitory profile was seen between the cultures of ZS-PA-35 + phipa2 and ZS-PA-16 + phipa4, except the growth suppression between phipa2-amended cultures and the controls were less evident in the first 5 h (Fig. 2). We hypothesize that the resurgence of growth in both phage cultures was probably a result of the regrowth of phage-resistant subpopulations.

### Phage mutation rate and phage-resistant physiological fingerprint.

Bacteria can defend against phage infection through various strategies, i.e., preventing phage entry, restriction-modification systems, abortive infection, and CRISPR-Cas systems (25–28). In order to understand the defense mechanisms underlying phage-host interactions, we first estimated the phage mutation rates of each phage-host population. Mutation rates of the phage phipa2, phipa4, and phipa10 mutants were calculated as ~2.61 × 10^−6^, ~4.43 × 10^−6^, and ~2.71 × 10^−6^, respectively. Thus, the mutation rate of strain ZS-PA-16 to phage phipa4 was higher than that observed of strain ZS-PA-35 to phage phipa2 and phipa10.

**FIG 3:**
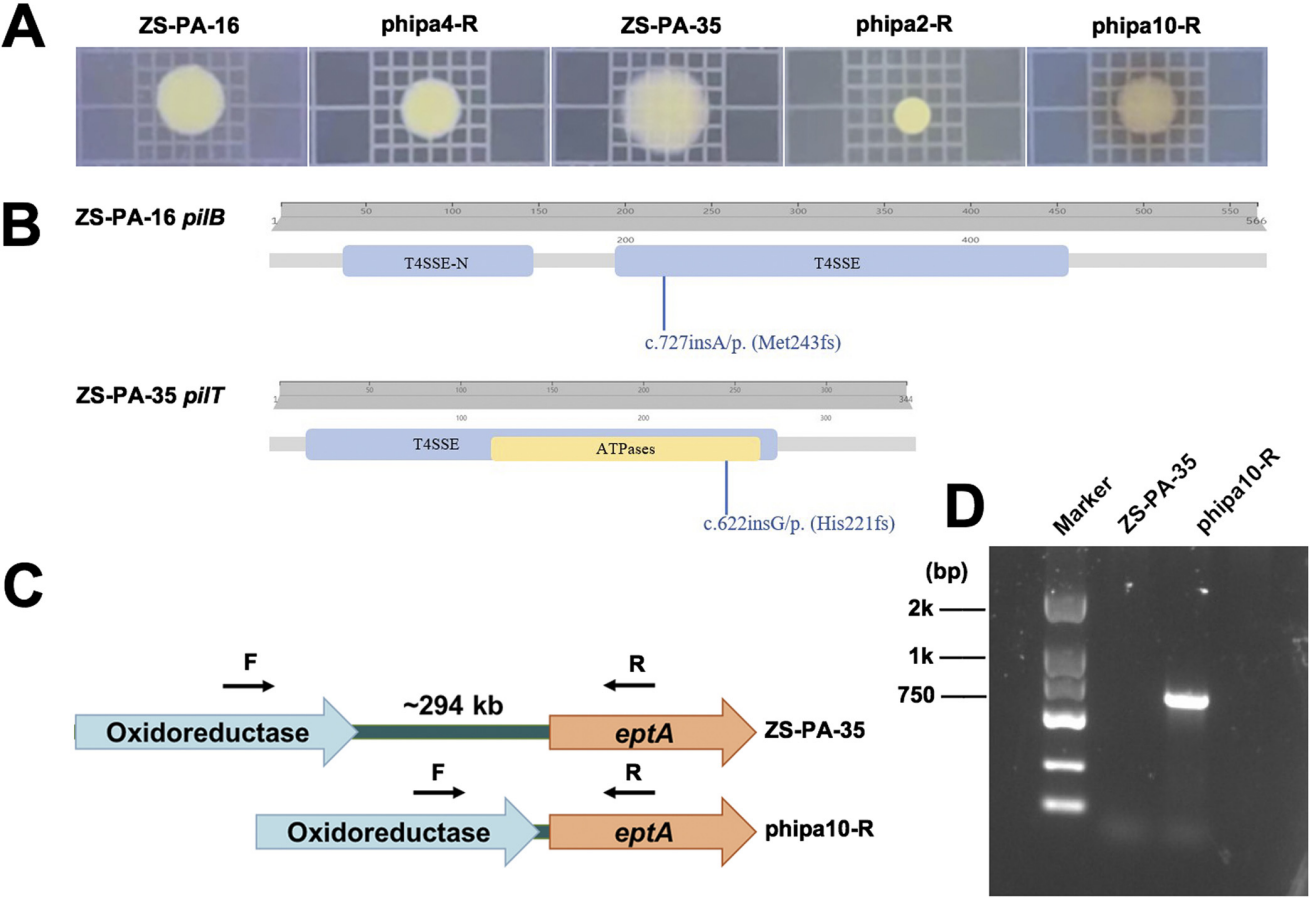
(A) Comparison of the bacterial colony morphology of wild-type strain and the corresponding phage-resistant mutants. (B) Comparative genomic analysis between the wild-type and phage-resistant mutants. Phage-resistant mutants were confirmed by PCR and Sanger sequencing. Schematic representation of frameshift insertion of G or A within gene *pilB* or *pilT* in phage-resistant mutant phipa2-R or phipa4-R is indicated in the wild-type functional domains. (C) Schematic representations of the location of primers are indicated on the flanking regions. Not to scale. (D) Agarose gel analysis of PCR product to detect the loss of ~294 kb chromosome fragment in strain ZS-PA-35 under phage phipa10 infection. The resulting band was confirmed by Sanger sequencing. EptA, lipid A phosphoethanolamine transferase.

Next, phage-resistant mutants derived from P. aeruginosa strains ZS-PA-16 and ZS-PA-35 were selected by confrontation with phipa2, phipa4, and phipa10. For each phage, out of 40 randomly picked surviving colonies were selected for the cross streak. More than ~40% of them were resistant to their corresponding phages. These resistant isolates were restreaked and screened by spot assay to confirm that they had acquired full phage resistance with heritable phenotype. Of these resistant isolates, 10 displayed full phage resistance (efficiency of plating, EOP = 0) and exhibited stable colony morphology variations with a small, transparent, and smooth phenotype. Among these fully resistant mutants, three representative phage-resistant mutants (denoted as “phipa2-R,” “phipa4-R,” and “phipa10-R”) were selected for downstream assays (Fig. 3A).

It is noteworthy that certain colony morphology uniquely emerged from specific phage treatments. For instance, a high percentage (~70%) of bacterial colonies from phipa10 lysate produced brown pigment in the surrounding agar after 24 h of incubation (Fig. 3A), indicating the overproduction of pyomelanin in strain ZS-PA-35 (32). In addition, a subpopulation (~30%) of small colony variants (SCVs) with distinguished morphology was constantly observed, and these SCVs were partially sensitive (i.e., reduced phage susceptibly compared to the parental strain) to phipa10. Altogether, these results reflect the phenotypic heterogeneities within P. aeruginosa phage-resistant mutants that enables subpopulation survival of fluctuating.

### Comparative genomics analysis of phage resistance.

To identify mutations associated with phage resistance, phage-resistant mutants were sequenced on an Illumina HiSeq platform, and assembled sequences were mapped to the reference genome of its parental P. aeruginosa strain. For phage-resistant mutant phipa2-R, a total of 125 mutations were identified. The effect impact of these mutations was categorized into four groups (high, 4/125; moderate, 18/125; low, 15/125; and modifier, 88/125) by SnpEff. For phage-resistant mutant phipa4-R, out of 74 mutations, seven were identified as high, the rest was categorized into moderate (12/74), low (12/74), and modifier (43/74). Mutations from the high effect group were likely to cause a phage-resistant phenotype, which was validated by PCR and Sanger sequencing. For phipa2-R mutant strains, a frameshift insertion of G in the position of 662 of gene *pilT* was identified, resulting in a histidine (H) to proline (P) substitution (Fig. 3B). This mutation possibly created a new protein with vastly different sequences compared to the wild-type sequence, and it may have inactivated the function of T4P. Likewise, a frameshift insertion of A in the position of 727 of gene *pilB* (567 aa) was observed in phage phipa4-R mutant, which yields a truncated protein (242 aa) (Fig. 3B). We speculate that the single nucleotide insertion in gene *pilB* identified in resistant strain phipa4-R results in amino acid changes due to a premature stop codon, possibly leading to phage insensitivity. Both PilT and PilB are predicted T4P secretion ATPases superfamily proteins, which are essential for extension and retraction. The motor ATPase PilB powers subunits pilin polymerization to form the pilus, whereas PilT is thought to interact with PilC (T4P pilus biogenesis protein) to facilitate depolymerization of the pilus fiber (29, 30).

The genetic basis of the resistance imposed by phage phipa10 was analyzed using Mauve. Results from genome comparisons confirmed that phipa10 infection caused an ~294 kb bp genomic fragment deletion in strain ZS-PA-35 (Fig. 3C). The deletion was confirmed by PCR and Sanger sequencing (Fig. 3D). Genomic analysis reveals that the deletion encompasses *galU* gene (UDP–glucose pyrophosphorylase) and *hmgA* gene (homogentisate 1,2-dioxygenase), which are known to be involved in LPS biosynthesis and to enhance the production of diffusible brown pigment pyomelanin (31, 32). Thus, we predicted that the loss of *galU* resulted in disruption of the bacterial cell wall surface integrity is associated with resistance to phage phipa10. These results are similar to previous studies showing that LPS serves as a receptor for most Pseudomonas phages (33).

**FIG 4:**
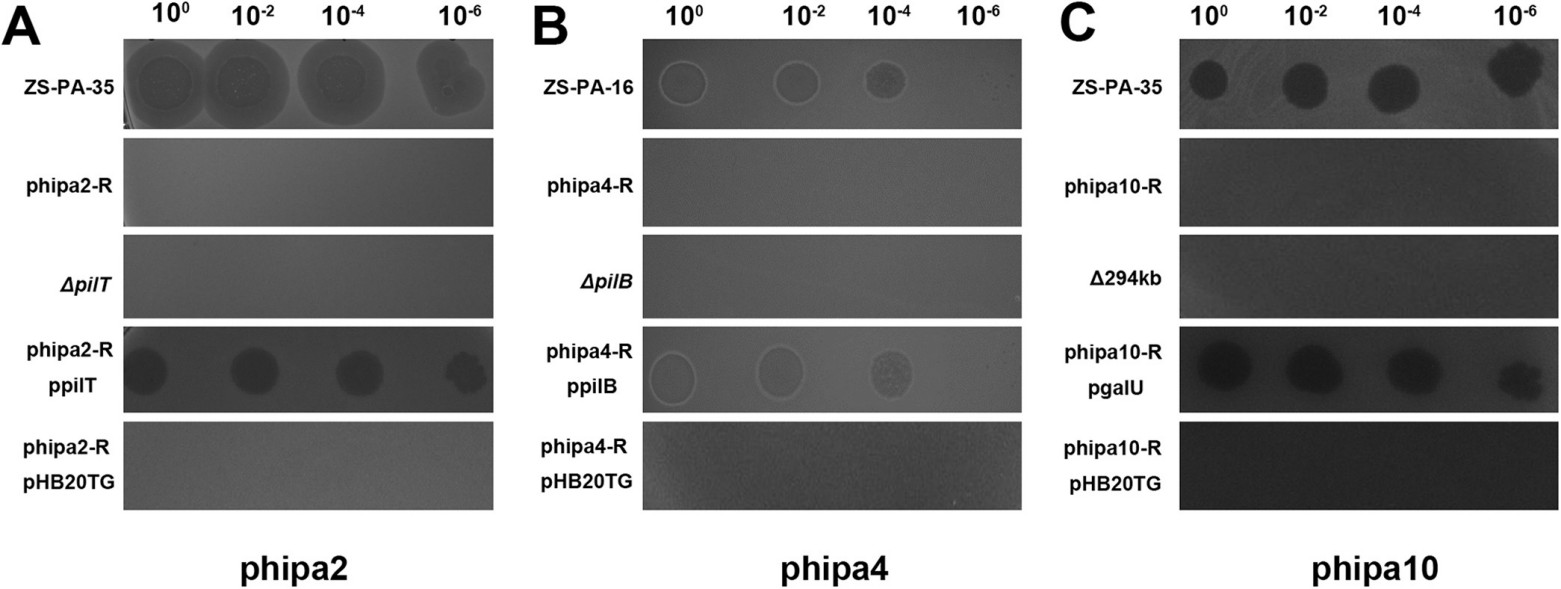
Spot test assay of phage phipa2 (A), phipa4 (B), and phipa10 (C) on the wild-type P. aeruginosa strains and their derived mutants. Lawns of the indicator strains were spotted with 100-fold serially diluted phage lysates. Phage-resistant mutants, in-frame deleted mutants, or phage-resistant mutants carrying empty plasmid pHB20TG failed to form plaques. Expression of gene *pilB*, *pilT*, or *galU* in phage-resistant mutants was induced with 0.4% arabinose and restored phage phipa2, phipa4, or phipa10 infectivity in the complementation assay.

To better understand the T4P and O-antigen as the potential phage receptors for phage phipa2, phipa4, and phipa10 infection in strains ZS-PA-16 and ZS-PA-35, three isogenic mutants Δ*pilB*, Δ*pilT*, and Δ294kb were constructed and screened for their efficiency of plating together with the wild-type strains and their derivatives. As expected, these in-frame deleted mutants were fully resistant to phage phipa2, phipa4, and phipa10 infections (Fig. 4). Also, phage susceptibilities of strain phipa4-R, phipa2-R, or phipa10-R were fully restored under the control of an arabinose-induced promoter directly on the plasmid pHB20TG with the expression of wild-type *pilB*, *pilT*, and *galU* genes (Fig. 4). In comparison, introduction of an empty pHB20TG plasmid in phage-resistant mutants phipa4-R, phipa2-R, and phipa10-R did not restore the phage infection nor lysis of the bacterial lawn (Fig. 4). Together, these findings not only agree with previous observations for T4P- and O-antigen-specific phages, but also provide supporting evidence into the role of the T4P and O-antigen of P. aeruginosa in phage-host interactions.

### The absence of gene *pilT* or *pilB* reduces motility and biofilm formation.

**FIG 5:**
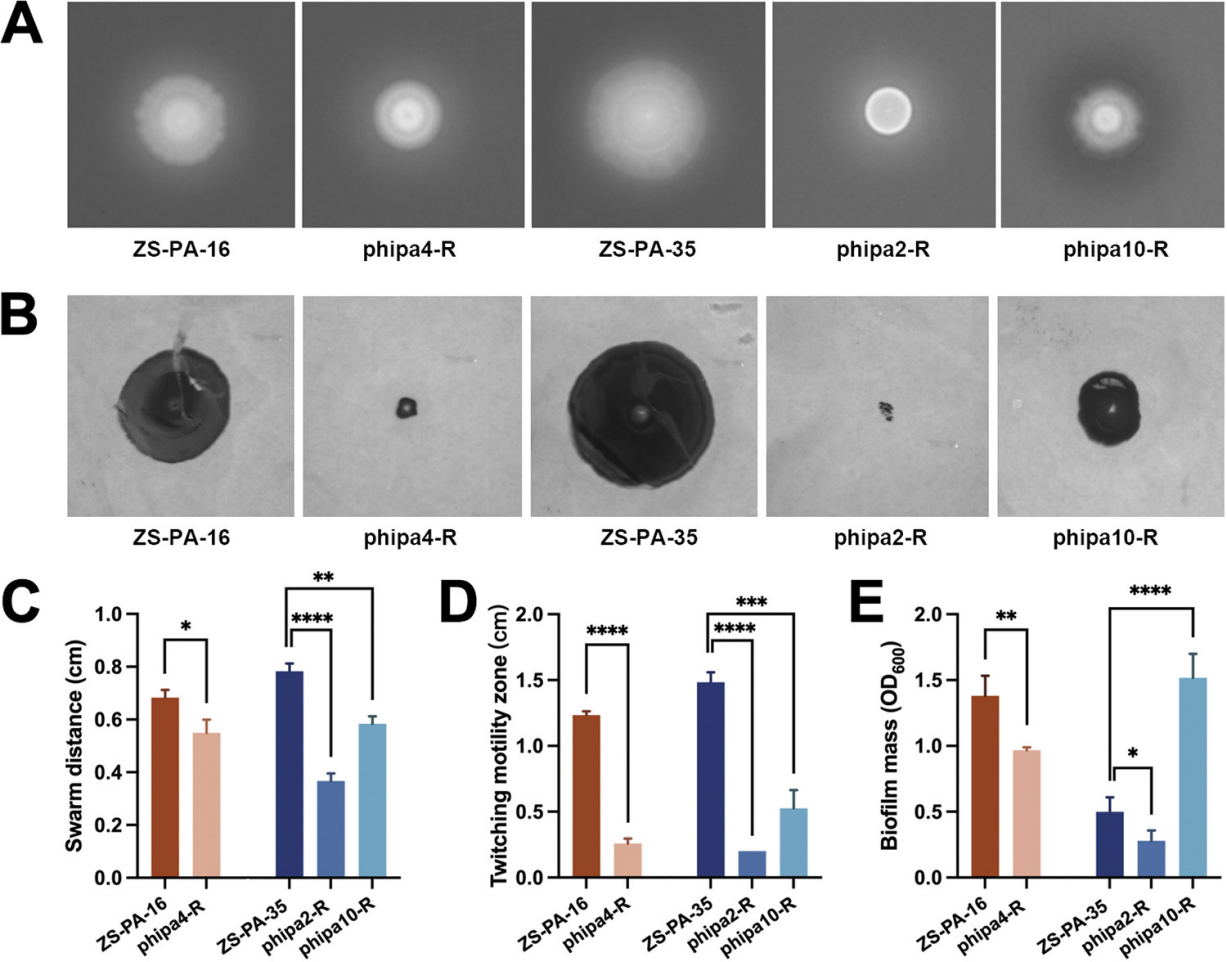
Roles of T4P and O-antigen in motilities and biofilm formation. (A) Examination of swarming motility of P. aeruginosa strains. Bacterial cells were inoculated at the center of 0.5% soft agar plates and incubated at 37°C for 72 h. (B) Examination of twitching motility of P. aeruginosa strains. Bacterial cultures were stabbed into LB agar plates (1.5%) and incubated at 37°C for 72 h. (C) Effect of phage resistance on the swarming motility, as determined by swarm distance (cm) of the cells. (D) Effect of phage resistance on the twitching motility, as determined by migration distance (cm) of the cells on the agar-petri dish interface. Quantification of swarming and twitching distances of the wild-type ZS-PA-16 and ZS-PA-35 and the resistant mutant strains was performed. For each strain, three individual plates were used for the analysis of the clone zones. (E) Biofilm formation in P. aeruginosa wild-type strains and phage-resistant mutants. Biofilm formation was stained by 0.4% crystal violet and quantified by absorbance. Error bars represent the standard deviations. All experiments were carried out in triplicate. Statistical analysis determined by Student's *t* test. *, *P* < 0.05; **, *P < *0.01; ***, *P < *0.001; and ****, *P < *0.0001.

To examine whether phage resistance is accompanied by fitness costs, we screened the wild-type and its one representative isolate for its swarming and twitching motilities. Strains of P. aeruginosa carrying a single mutation in gene *pilT* (*P* < 0.0001) or *pilB* (*P* < 0.05) exhibited marked decreases in swarming motility in LB soft agar (0.5%) compared to wild-type strains (Fig. 5A and C). In strain ZS-PA-35, the effect of *pilT* on swarming motility was even more prominent, with an ~53.2% decrease in the distance migrated. In contrast, mutations in gene *pilB* had a relatively light effect on motility, corresponding to only an ~19.5% decrease compared with wild-type strains (Fig. 5A and C). Next, the subsurface stab assay demonstrated that phage-resistant mutants (phipa2-R and phipa4-R) were completely defective in twitching mobility (*P* < 0.0001) (Fig. 5B and D). This suggests that phage selection-mediated loss of T4P function affects both swarming and twitching motilities (34, 35). Bacterial motility has been linked to biofilm morphology and spreading dynamics. Next, we sought to quantify the biofilm formation of the wild-type strains and phage-resistant mutants over a long-term experiment. In keeping with the motility observed for the phage-resistant mutants, biofilm formation was remarkably reduced in phage-resistant mutant strains phipa2-R (*P* < 0.05) and phipa4-R (*P* < 0.01) (Fig. 5E).

### Large chromosomal deletion strain shows impaired motility and promotes biofilm formation.

Current epidemiology data indicate that there are approximately 20 different serogroups in P. aeruginosa, and are their diversities are reflected in monosaccharide structures between sugar residues (36). Previous studies revealed that a mutation in gene *galU* resulted in a loss of LPS conferring phage resistance and attenuated virulence (31, 37). To analyze the role of large chromosomal deletion (~294 kb) in motility and biofilm formation, we first confirmed that the deletion did not affect the growth rates (data not shown). Focusing on motility-related phenotypes, we found that phipa10-R mutant had decreased both swarming (*P* < 0.01) and twitching (*P* < 0.001) motilities on soft agar and surface-associated movement (Fig. 5). In addition, we analyzed biofilm formation by wild-type strain ZS-PA-35 and phage phipa10 resistant mutant. Unexpectedly, the amount of biofilm biomass of phage phipa10-R mutant was 3 times higher than that of the wild-type strain (*P* < 0.0001) (Fig. 5E). These results demonstrate that the large chromosomal deletion limits bacterial motility, while simultaneously promoting biofilm formation to create a less disadvantageous phenotype.

### Phage selection mediated changes in antibiotic susceptibility.

**FIG 6:**
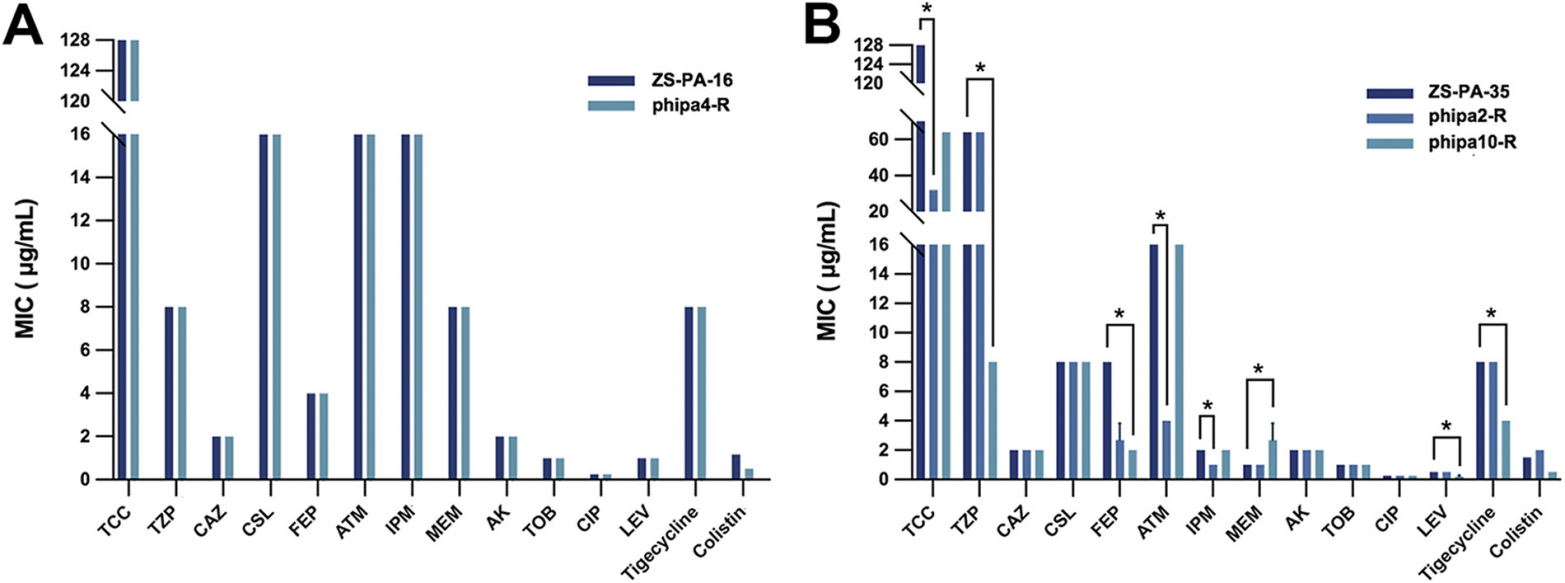
Antibiotic susceptibilities of wild-type strains and phage-resistant mutants were determined by Vitek 2. Changes in MIC of the phage-resistant mutants compared to wild-type strains were represented in *y* axis (μg/mL). (A) Selection of phage phipa4 did not affect antibiotic sensitivity in strain ZS-PA-16. (B) Selection of phage phipa2 or phipa10 restored certain antibiotic sensitivities in strain ZS-PA-35. All experiments were performed in triplicate. Repeated differences were evaluated using the Kruskal-Wallis H test and *post hoc* Dunn test, *, *P* < 0.05. TCC, ticarcillin/clavulanic acid; TZP, piperacillin-tazobactam; CAZ, ceftazidime; CSL, cefoperazone/sulbactam; FEP, cefepime; ATM, aztreonam; IPM, imipenem; MEM, meropenem; AK, amikacin; TOB, tobramycin; CIP, ciprofloxacin; LEV, levofloxacin.

To determine whether the phage-resistant mutants altered their antibiotic susceptibilities, i.e., increased antibiotic sensitivity via antagonist pleiotropy, we performed their MICs for 14 clinically relevant antibiotics using the Vitek 2 system. As shown in Fig. 6A, wild-type strain ZS-PA-16 and phage-resistant mutant phipa4-R exhibited identical antibiotic susceptibility test (AST) profiles. In contrast, phage-resistant mutants (phipa2-R and phipa10-R) showed different AST profiles than that of wild-type strain ZS-PA-35 (Fig. 6B). Of 14 antibiotics analyzed, resistant to ticarcillin-clavulanic acid and tigecycline, susceptible to ceftazidime, cefoperazone/sulbactam, cefepime, amikacin, tobramycin, ciprofloxacin, levofloxacin, and colistin; and intermediate to aztreonam were observed in both strain ZS-PA-16 and ZS-PA-35. Following the observed fitness costs incurred by phage resistance, phage-resistant mutant phipa2-R increased their susceptibilities of ticarcillin-clavulanic acid, aztreonam, and imipenem dramatically from resistant (≥128 μg/mL) to intermediate (32 μg/mL), intermediate (16 μg/mL) to sensitive (4 μg/mL), and sensitive (2 μg/mL) to sensitive (1 μg/mL), respectively (*P* < 0.05) (Fig. 6B). In line with the results from phage phipa2, the selection of phage phipa10 significantly decreased its resistance to piperacillin-tazobactam by 8-fold (64 μg/mL to 8 μg/mL), cefepime by 4-fold (8 μg/mL to 2 μg/mL), levofloxacin by 2.4-fold (0.5 μg/mL to ~0.21 μg/mL), and tigecycline by 2-fold (≥8 μg/mL to 4 μg/mL) (*P* < 0.05). Unexpectedly, phage phipa10-R resistant mutant conferred increased resistance to meropenem slightly, shifting from the MIC from 1 μg/mL to ~2.6 μg/mL (*P* < 0.05) (Fig. 6B). Together, these results indicate that phage resistance mutations in genes related to T4P or O-antigen biosynthesis would result in changes in antibiotic susceptibilities, highlighting that synergistic phage-antibiotic combinations may offer a promising strategy to reduce the dose during treatment. However, such applications need to unravel the unexpected complexity of phage-host evolution.

## DISCUSSION

The genetic disorder cystic fibrosis increases one’s susceptibility to chronic infections and these infections are often recalcitrant to traditional antibiotics. With the rise of MDR bacterial pathogens, phage therapy as an alternative or supplement to antibiotics has become ever important due to their strong lysis and specificity. In this study, we isolated and characterized three novel phages (phipa2, phipa4, and phipa10) against clinical isolates of P. aeruginosa. Our genomic analyses show that all phages show no clear sequence similarities to each other, despite being isolated from the same sewage source. In addition, we observed a unique sequence in phage phipa4 encoded *mazG* gene, having a putative nucleoside triphosphate pyrophosphohydrolase previously implicated in regulation of type II MazE-MazF toxin-antitoxin (TA) system via lowing cellular concentration of (p)ppGpp under stress conditions, such as amino acids or energy starvation (38). With this finding, we hypothesize that phage phipa4 might bring auxiliary metabolic genes to the host to optimize the cellular physiological status for phage replication. Moreover, phage phipa10 is shown to possess 11 tRNAs (Gln, Arg, Lys, Leu, Ile, Asp, Cys, Pro, Gly, Phe, and Glu), most likely corresponding to codons that are highly used by phage translation to improve its virulence and fitness. Thus, deciphering the mechanisms governing phage-host interactions will provide us with better understanding of the impact of bacterial phenotypic and genetic response to phage infection.

### Mutations associated with phage resistance linked to T4P and O-antigen.

In phage therapy, repeated phage dosing is recommended to increase phage concentration at sites of infections. However, previous studies have shown that high dosages of phage and long-duration treatments are more likely to select phage-resistant mutants (39). Generally, bacteria are subject to constant phage selection pressures, and could lead to antagonistic coevolution between hosts and phages (40). Coevolution is characterized by the rapid evolution of genetic traits and genetic diversity following the initial lysis, which has the potential to influence phage-host interactions (41). In this study, we fully sequenced phage-resistant mutant strains of P. aeruginosa and explored the selected mutants through comparative genomic analysis. We show that the phage-resistant mutants carried impairing function of proteins involved in T4P assembly or LPS O-antigen biosynthesis. These two main predicted cell surface receptors have been found to be common binding sites for P. aeruginosa phages, and it has been previously demonstrated that mutations of subunits T4P and O-antigen caused cell surface alterations that blocked phage adsorption and subsequent cell lysis (33). Unfortunately, no other mutations were observed in pilus-related genes from the phage-resistant mutants in this study, only two of the pilus-related genes were identified, covering only a small fraction of T4P biosynthesis and function in P. aeruginosa (42). Other mutations in T4P structural genes, such as *pile*, *pilV*, *pilY1*, and *fimV*, or regulatory genes, *pilJ*, *pilR*, *pilS*, and *algR*, observed in P. aeruginosa transposon mutants have also conferred resistance to phages (19). Undoubtedly, repetitive regions may exist in reference assembly, which may not be reconstructed and missing, can fragment assemblies, and adversely impact downstream analyses (43). For the lytic phage phipa10, phage-resistant mutant phipa10-R was accompanied by the loss of a large genome section carrying *galU*, known as the requirement for a complete lipopolysaccharide core (31). Therefore, we inferred T4P to be the phage receptor for phipa2 and phipa4, and O-antigen to be the phage receptor for phipa10. In addition, these phage-resistant mutations exhibited some pleiotropic effects on the host fitness that involved motility, biofilm formation, and antibiotic susceptibility, emphasizing the role of phage-driven genetic and phenotypic diversification in P. aeruginosa.

### Mutations have different effects on host fitness.

Biofilm is an assemblage of surface-associated cells enclosed in a matrix, which is made up of extracellular polymeric substances (EPS), extracellular DNA (eDNA), and cell debris (44). As an important element of pathogenicity, the formation of biofilms by P. aeruginosa poses a serious threat to human health care due to its strong ability to colonize biomaterials and its resistance to antimicrobial agents (45). Some phages have been studied and proven to be effective in eradicating biofilms due to their production of depolymerase (46). Yet, others also showed that phages can promote the formation of biofilms to promote the coexistence of phage and its hosts under certain conditions, such as low environmental pH (47, 48). Thus, it is becoming increasingly important to study phage-host interactions in the context of biofilm formation. We demonstrated that phage phipa2, phipa4, and phipa10 played important roles in the transition between planktonic growth and biofilm development by replacing the sensitive population with subpopulations of phage-resistant mutants, carrying gene mutations in either *pilT*, *pilB* or *galU*. Both T4P and O-antigen have been shown to regulate key phenotypic traits of biofilm formation and motility by diverse mechanisms. Within P. aeruginosa studies, there are examples of both O-antigen- and T4P-activated or repressed biofilm formation. For example, P. aeruginosa PAO1 strain deficient in *galU* was significantly attenuated in the formation of biofilms, indicating that the absence of the O-side chains on the bacterial surface affects biofilm formation. Likewise, excess twitching can impair biofilm formation in the initial phase, showing that the iron-chelating protein lactoferrin increased twitching motility with reduced biofilm formation (49). However, the phenotypes of phage-resistant mutant phipa10-R suggested the opposite, namely, that *galU*-mediated O-antigen defective in strain ZS-PA-35 significantly increased biofilm formation through enhanced surface attachment; while in the phage-resistant mutants (phipa2-R and phipa4-R) lacking the functional T4P, biofilm formation was significantly reduced compared to those of controls. Thus, large intraspecific variations existed in biofilm formations between different P. aeruginosa strains under phage predation. Taken together, our results are in line with previous studies that phages provide additional important roles in regulating biofilm formation in P. aeruginosa, and some of those symbiotic relationships can be established to induce and strengthen biofilms.

### Phage resistance restores antibiotic sensitivity.

In addition to the roles of T4P and O-antigen in phage receptors and biofilm formation, we investigated the potential evolutionary interactions between antibiotic susceptibility and phage resistance. As expected, we found that phage-resistant mutants restored certain antibiotic sensitivities. Our finding supports the previous study that phage selection imposes strong trade-offs between phage resistance and antibiotic sensitivity. For instance, Pseudomonas phage OMKO1 adsorbs to the outer membrane porin M (OprM) of the MexAB and MexXY multidrug efflux systems, and in doing so selects for phage resistance and antibiotic sensitivity (50). However, for phipa2 and phipa10, the mechanism responsible for changes in phage resistance-mediated antibiotic susceptibility has not been determined and requires further studies. However, it is thought that T4P might help the bacterial host to alter collective motion to avoid antimicrobial compounds, as it has been demonstrated that the Δ*pilA* mutant was much more susceptible to a pulmonary innate immunity surfactant protein-A (SP-A)-mediated bacterial opsonization and membrane permeabilization (51). For the large fragment loss mutation in strain ZS-PA-35, the phage selected mutation in *galU* gene led to the truncation of the LPS core, presumably increasing the permeability of the cell envelope for hydrophilic antibiotics (52). Similarly, the susceptibility of a Streptococcus pneumoniae *galU* mutant R6 increased over 100-fold toward β-lactam antibiotics amoxicillin and cefuroxime (53). These studies raise the possibility to reduce the dose of noneffective antibiotics and the development of antibiotic resistance. For instance, application of phage therapy in combination with antibiotics has been successfully explored in treating drug-resistant K. pneumoniae in clinical trial studies (54).

### Implications for phage therapy.

Using different phage-host interaction systems, we provide solid fundamental knowledge for potential applications of phage therapy. Nevertheless, it should be noted that all variations of the phenotypic traits were observed under highly uniform laboratory conditions, while fitness trade-offs resulting from phage resistance may be even more complex and broad *in vivo* (55–57). For instance, artificial sputum is more appropriate to mimic the lung infection of cystic fibrosis patients and to study the coevolution between bacteria and phage in conditions more relevant. Moreover, genetic mutations other than those resolved by the approach may have been present, as a limited number of phage-resistant mutants were selected for the follow-up study. Consequently, more work remains to be done to elucidate the interplay of phage-host and the impact of resistant evolution on bacterial fitness to increase our abilities to implement new phage technologies and therapies in combination with conventional antimicrobials.

## MATERIALS AND METHODS

### Bacterial strains and plasmids.

Strains, plasmids, and phages used in this study are listed in Table S1. Bacterial isolates used in this study were originally isolated from Zhongshan Hospital, Shanghai, China. Strains were grown at 37°C in LB Miller broth (containing peptone 10 g/L, yeast extract 5 g/L, and NaCl 10 g/L) or on 1.5% LB agar plates under aerobic conditions. Antibiotics were added at the following concentrations: 30 μg/mL gentamicin and 100 μg/mL irgasan for P. aeruginosa and 15 μg/mL gentamicin for Escherichia coli.

### DNA manipulation and complementation of pilus mutants.

To construct chromosome in-frame deletion of *pilB* and *pilT* genes in P. aeruginosa ZS-PA-16 and ZS-PA-35, DNA fragments flanking the gene of interest were PCR amplified from the wild-type strain using primers to introduce XbaI and XhoI sites and overlap of identical sequence (~20 bp). The upstream and downstream DNA sequences were used as the template for an overlap extension PCR. The PCR product was digested with XbaI and XhoI FastDigest Restriction Enzymes (Thermo Fisher Scientific, CA, USA) and cloned into the corresponding sites of plasmid pEXG2 (58). The resulting plasmids were transferred into E. coli SM10 using the heat shock method, and subsequently conjugated into P. aeruginosa strains. Transconjugants were first selected on LB agar plates containing gentamicin and irgasan. All the inserted mutants were recovered by incubating in low-nutrient 1.5% agar plates (peptone 5 g/L, yeast extract 1 g/L, and NaCl 5 g/L) containing 5% (wt/vol) sucrose at room temperature for 24 h. Desired mutants were identified by PCR and verified by sequencing. The Δ294kb deletion plasmid, pEXG2-294, was constructed as described above, except the flanking regions were directly amplified from the template of phage phipa10-R mutant gDNA. All primers used are listed in Table S2.

The *pilB*, *pilT*, and *galU* genes were amplified from P. aeruginosa genomic DNAs by PCR. The resulting products were double digested with XbaI + EcoRI, XbaI + HindIII, or XbaI + EcoRI FastDigest Restriction Enzymes (Thermo Fisher Scientific, CA, USA) and ligated (T4 DNA ligase, Thermo Fisher Scientific, CA, USA) into the vector pHB20TG. The resulting plasmids (designed ppilB, ppilT, and pgalU) were verified by Sanger sequencing and transferred into E. coli competent SM10 cells before mobilizing into the phage-resistant mutant strain phipa4-R, phipa2-R, or phipa10-R via bacterial conjugation as described above. Transformants that acquired a recombinant plasmid or the empty vector were screened for EOP (efficiency of plating) on the double-layer LB plates supplemented with 0.4% L-arabinose (Sigma-Aldrich, St. Louis, MO, USA) to induce the pBAD promoter.

### Phage isolation and purification.

Sewage samples were collected from Zhongshan Hospital (Shanghai, China). Sample aliquots (100 mL) were incubated with 100 mL of 2 × LB liquid medium and 1 mL mid-log-phase P. aeruginosa strain for ~6 h at 37°C. Enrichments were centrifuged at 4°C at 8,000 × *g* for 5 min. Cell-free supernatants were sterilized by filtration (0.22 μm, Millipore, MA, USA). Phage was detected by spotting 5 μL of the sterilized enrichment onto 0.5% LB top agar inoculated with mid-log-phase P. aeruginosa. Individual phages were purified until their plaque morphologies were homogeneous by plaque assay. Using strain ZS-PA-16 and ZS-PA-35, phage phipa2, phipa4, and phipa10 were amplified to generate high titer phage lysate and stored at 4°C.

### Phage morphology analysis.

Phage lysates were serially diluted in PBS to a titer of approximately 10^8^ PFU/mL to reduce background. Formvar/carbon-coated copper grids (200 mesh, Sigma-Aldrich, St. Louis, MO, USA) were incubated with diluted phage lysates for 5 min to allow adsorption. Grids were negatively stained with 2% (wt/vol) sodium phosphotungstate for 2 min. Grids were washed in a drop of distilled water and excess liquid was removed by filter paper and then air-dried prior to being visualized using a JEM-2100 microscope (Hitachi, Japan) operated at 80 kV. Image analysis was performed using ImageJ software (https://imagej.nih.gov/ij/).

### Phage-host interaction in liquid LB broth.

The susceptibilities of strains ZS-PA-16 and ZS-PA-35 to their corresponding phage were analyzed by the measurement of bacterial optical density at 600 nm. Briefly, overnight (O/N) strains of ZS-PA-16 and ZS-PA-35 were 1,000-fold back diluted in 15 mL of LB broth and grown to an OD of ~0.15, corresponding to 1.2 × 10^8^ CFU/mL. Phages were added at the MOI of 2 in triplicate, and with parallel controls without phages. The effect of phage-lysis on host cells was monitored every hour over the 6-h incubation.

### Bacterial and phage genomic DNA extraction.

Bacterial genomic DNA was extracted using Wizard Genomic DNA purification kit (Promega, Madison, WI, USA) according to the manufacturer’s protocol. The quality of each extracted DNA sample was verified using a Nanodrop and agarose gel electrophoresis. Bacterial genomes were sent for Illumina sequencing at Sangon Biotech (Sangon, Shanghai, China). Phage DNA was extracted using the Qiagen DNeasy blood and tissue kit (Qiagen, CA, USA) according to the manufacturer’s protocol. In brief, residual bacterial genomic DNA and RNA were removed from phage lysates following digestion with DNase I and RNase A (Thermo Scientific, CA, USA) for 1.5 h at 37°C (59). DNase I and RNase A were inactive by EDTA (final concentration 20 mM) and heating at 80°C for 5 min, after which the manufacturer’s instructions were followed. Sequencing was done at the Chinese National Human Genome Center (Shanghai, China) using an Illumina platform.

The raw sequence read quality was checked by using FastQC V0.11.8 (http://www.bioinformatics.babraham.ac.uk/projects/fastqc), and then trimmer and filtered by using Trimmomatic V0.39 (http://www.usadellab.org/cms/?page=trimmomatic) (60). Genomes were assembled using SPAdes v3.5.0 (http://cab.spbu.ru/software/spades/) (61). Gaps with contigs were closed using GapFiller V1.11 (https://www.baseclear.com/genomics/bioinformatics/basetools/gapfiller) (62). Sequencing errors were corrected using PrInSeS-G V1.0.0 (https://updeplasrv1.epfl.ch/prinses/) (63). Both bacterial and phage genomes were annotated using RAST (https://rast.nmpdr.org/) (64). Visualization of phage genomes were produced by Proksee (https://proksee.ca/).

### Isolation of phage-resistant mutants and comparative genomic analysis.

Phage-resistant mutants were isolated as described previously (65). Briefly, O/N bacteria were 1,000-fold back diluted in LB broth and incubated at 37°C aerobically to an OD600 of 0.3. Aliquots (1 mL) of the bacterial culture were mixed with high titer phage lysate at an MOI of 10. The mixture was incubated at 37°C for 10 min and inoculated onto LB agar plates. After incubating O/N at 37°C, the number of colonies on each plate was counted to calculate phage mutation rates. All experiments were conducted in triplicate. The resistance phenotype of surviving colonies was checked by cross-streak assays, and further purified and confirmed by spot test. Phage-resistant mutants gDNA were extracted as described above and sequenced using the Illumina Hiseq platform (Sangon Biotech, Shanghai, China) to determine genetic changes. Briefly, low-divergent phage-resistant reads were mapped to the reference genome (wild-type strain ZS-PA-16 and ZS-PA-35) by using Burrows–Wheeler Aligner (BWA V0.7.17, http://bio-bwa.sourceforge.net/) and the Genome Analysis Toolkit (GATK V4.1.1.0, https://gatk.broadinstitute.org/hc/en-us) best practices pipeline (66, 67). Single nucleotide polymorphisms (SNPs), including base substitutions, deletions, and insertions, were filtered using MarkDuplicates (https://gatk.broadinstitute.org/hc/en-us/articles/360037052812-MarkDuplicates-Picard-). Read depth for each gene was analyzed using BEDTools V2.28.0 (https://bedtools.readthedocs.io/en/latest/) (68). SNP effects on genes were predicted by SnpEff V4.3T (https://pcingola.github.io/SnpEff/) and their impacts were categorized as high, moderate, low, or modifier (69).

Unfortunately, PCR and Sanger sequencing failed to validate SNPs between wild-type strain ZS-PA-35 and phage-resistant mutants phipa10-R as described above. Subsequent draft genome alignment was performed to detect any chromosomal fragment losses in strain phipa10-R with the reference sequence of ZS-PA-35 and PAO1 as templates by Mauve V2.3.1 (https://darlinglab.org/mauve/mauve.html) (70).

### Swarming and twitching motility and biofilm formation assays.

To quantify the functional properties of the different strain variants, motility and biofilm assays were applied. For the swarming motility assay, O/N bacteria were 100-fold diluted and grown to an exponential phase with an OD600 nm of 1.0. Aliquots (0.2 μL) of bacteria were spotted on LB agar (0.5%) plates and incubated at 37°C for 72 h. Swarming motility was determined by measuring the distance of the spreading colony.

Twitching motility assay was performed according to the method described by Shreeram et al. with slight modifications (71). Briefly, aliquots (2.5 μL) of O/N bacterial cultures were stabbed into the center bottom of the petri dish through 1.5% LB agar. After incubating at 37°C for 72 h, the agar was removed and the zone of motility of each plate was visualized by staining with 0.4% crystal violet for 30 min statically at room temperature. Excess stain was removed by washing in tap water. Twitching motility was determined by measuring the distance of the solid surface translocation on petri dish plates.

Biofilm formation analysis was performed in a 10-mL polystyrene tube as described previously with crystal violet staining (72). Briefly, a 5 μL aliquot of O/N bacterial culture was inoculated in 5 mL LB broth and incubated statically to develop a mature biofilm. After 10 days, the liquid was removed and each tube was stained with 4 mL of 0.4% crystal violet solution. Excess stain was removed by triplicate washings with tap water. Biofilms were evaluated by adding 4 mL of 33% acetic acid to each tube, allowing the stain to dissolve and then be quantified by their absorbance at OD600 nm. All experiments were performed in triplicate.

### Antibiotic susceptibility assay.

Antimicrobial susceptibilities of P. aeruginosa wild-type strains and phage-resistant mutants were performed following Vitek 2 (bioMérieux, France) instructions. Phage-resistant mutants were isolated as described above. A Vitek susceptibility card (AST-N335 for Gram-negative bacteria) was used for each isolate, including 14 commonly antibiotics (ticarcillin-clavulanic acid, piperacillin-tazobactam, ceftazidime, cefoperazone/sulbactam, cefepime, aztreonam, imipenem, meropenem, amikacin, tobramycin, ciprofloxacin, levofloxacin, tigecycline, and colistin), according to the manufacturer's instructions. All experiments were performed in triplicate.

### Statistical analysis.

Statistical analyses and generations of the graphs were performed using GraphPad Prism 9.2 (GraphPad, La Jolla, CA). All data are expressed as the mean ± SD. Student's *t* test or a two-way ANOVA was used to compare the swarming motility, twitching motility, and biofilm formation between wild-type strains and phage-resistant mutants. Differences in antibiotic sensitivity between wild-type strains and phage-resistant mutants were analyzed using the Kruskal–Wallis test with Dunn *post hoc* tests. A *P*-value < 0.05 was considered as significant differences.

### Data availability.

The genomes of P. aeruginosa have been deposited in GenBank under the BioProject accession PRJNA771935, with the following accession numbers: ZS-PA-16 (GCA_020567465.1) and ZS-PA-35 (GCA_020567355.1). Phage genomes were deposited in GenBank under the following accession numbers: phipa2 (OK539824.1), phipa4 (OK539825.1), and phipa10 (OK539826.1).
