# Supplementary material for: Characterization of Phage Resistance and Their Impacts on Bacterial Fitness in Pseudomonas aeruginosa
Source: Microbiol Spectr. 2022 Sep 21;10(5):e02072-22. doi: 10.1128/spectrum.02072-22 (PMC9603268; doi:10.1128/spectrum.02072-22)
Supplement: Supplemental file 1 — Table S1, Table S2, Fig. S1. Download spectrum.02072-22-s0001.pdf, PDF file, 0.6 MB [file spectrum.02072-22-s0001.pdf]

Table S1. Bacterial strains, plasmids, and bacteriophages.

Table S2. Oligonucleotides used in this study.

FIG S1. Schematic maps of phage genomes (phipa2, phipa4, and phipa10).

Name	Genotype or relevant markers
ZS-PA-16	wildtype, *Pseudomonas aeruginosa*, host for phipa4
ZS-PA-35	wildtype, *Pseudomonas aeruginosa*, host for phipa2 and phipa10
Δ*pilB*	Clean deletion of *pilB*, *Pseudomonas aeruginosa*
Δ*pilT*	Clean deletion of *pilT*, *Pseudomonas aeruginosa*
Δ294	Clean deletion of 294, *Pseudomonas aeruginosa*
SM10	λ-pir, Escherichia *coli*
pEXG2	Allelic exchange vector with pBR origin, Gm^R, *sacB*
pdeltapilB	pEXG2, carrying *pilB* flanking regions, Gm^R
pdeltapilB	pEXG2, carrying pilT flanking regions, Gm^R
pdelta294kb	pEXG2, carrying 294kb flanking regions, Gm^R
pHB20TG	Arabinose-inducible pBAD promoter, Gm^R
ppilB	pHB20TG carrying ZS-PA-16 *pilB*, Gm^R
ppilT	pHB20TG carrying ZS-PA-35 *pilT*, Gm^R
pgalU	pHB20TG carrying ZS-PA-35 *galU*, Gm^R
phipa2	*Podoviridae*, 43 kb, lytic phage
phipa4	*Siphoviridae*, 42 kb, lytic phage
phipa10	*Myoviridae*, 92 kb, lytic phage

Name	Primers (5’-3’)
pilB_1	GTGCCAAGCTTATTTGCGGTAGC
pilB_2	GCTTTGTCCGCCATGGAGGGGAAGGAATCGCAGAAGGGCT
pilB_3	CTGCGATTCCTTCCCCTCCATGGCGGACAAAGCGTTAAAAACCAGCG
pilB_4	TTTTCTAGAGTTTTCCAAGGCGCCCGATTG
pilT_1	TTTTCTAGACCAAGGTTCAGGTCCAGCAG
pilT_2	ATCGGCGCCAGGAGGGACTCCCCAATTACAAGC
pilT_3	GGAGTCCCTCCTGGCGCCGATCCGCCGCGCTTCGCCCGAATC
pilT_4	TTTTCTAGAGGTTGCGCTGGTAGAAGGC
294kb_1	TTTTCTAGAGACCGGTCATGAATCGAGCAAC
294kb_2	TTTAAGCTTATGTCGAAAGCCCGTGCCGT
pilB_com_1	TTTGAATTCATGAACGACAGCATCCAACTG
pilB_com_2	TTTTCTAGATTAATCCTTGGTCACGCGGTTG
pilT_com_1	TTTTCTAGAATGGATATTACCGAGCTGCTCGCC
pilT_com_2	TTTAAGCTTTCAGAAGTTTTCCGGGATCTTCGCC
galU_com_1	TTTGAATTCATGATCAAGAAATGTCTTTTCC
galU_com_2	TTTTCTAGATCAGTGAGCCTTGCCGGTC

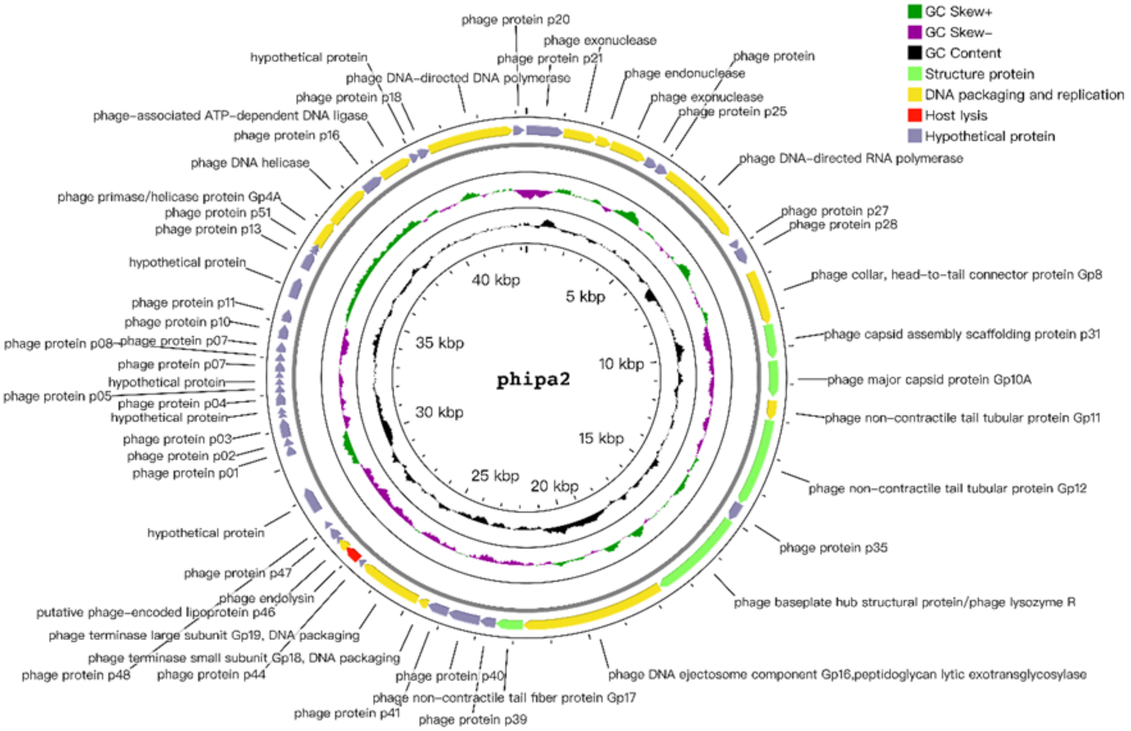
GC Skew+
GC Skew−
GC Content
Structure protein
DNA packaging and replication
Host lysis
Hypothetical protein
phipa2
40 kbp
5 kbp
35 kbp
10 kbp
30 kbp
15 kbp
25 kbp
20 kbp
phage protein p20
phage exonuclease
hypothetical protein
phage protein p21
phage protein
phage DNA-directed DNA polymerase
phage endonuclease
phage protein p18
phage exonuclease
phage-associated ATP-dependent DNA ligase
phage protein p25
phage protein p16
phage DNA-directed RNA polymerase
phage DNA helicase
phage primase/helicase protein Gp4A
phage protein p51
phage protein p27
phage protein p13
phage protein p28
hypothetical protein
phage collar, head-to-tail connector protein Gp8
phage protein p11
phage protein p10
phage protein p08
phage protein p07
phage capsid assembly scaffolding protein p31
phage protein p07
hypothetical protein
phage major capsid protein Gp10A
phage protein p05
phage protein p04
hypothetical protein
phage non-contractile tail tubular protein Gp11
phage protein p03
phage protein p02
phage protein p01
phage non-contractile tail tubular protein Gp12
hypothetical protein
phage protein p35
phage protein p47
phage endolysin
phage baseplate hub structural protein/phage lysozyme R
putative phage-encoded lipoprotein p46
phage terminase large subunit Gp19, DNA packaging
phage terminase small subunit Gp18, DNA packaging
phage protein p48
phage protein p44
phage protein p40
phage DNA ejectosome component Gp16,peptidoglycan lytic exotransglycosylase
phage non-contractile tail fiber protein Gp17
phage protein p41
phage protein p39

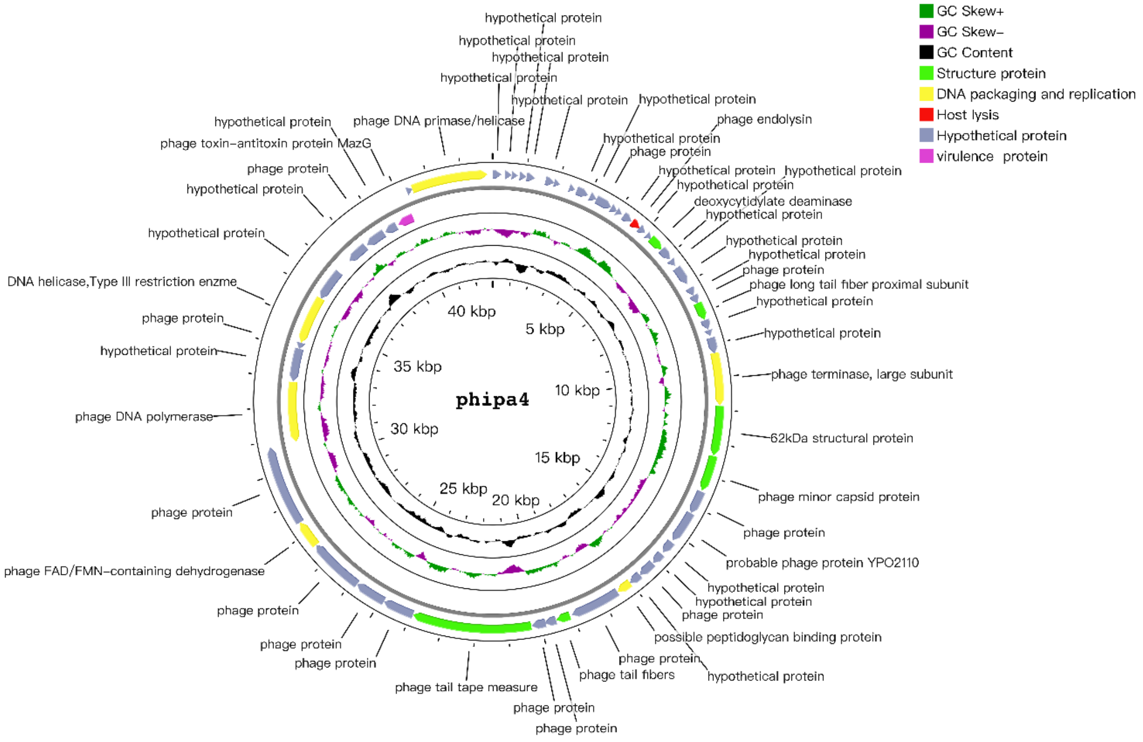
GC Skew+
GC Skew−
GC Content
Structure protein
DNA packaging and replication
Host lysis
Hypothetical protein
virulence protein
phipa4
40 kbp
5 kbp
35 kbp
10 kbp
30 kbp
15 kbp
25 kbp
20 kbp
hypothetical protein
hypothetical protein
hypothetical protein
hypothetical protein
hypothetical protein
hypothetical protein
hypothetical protein
phage DNA primase/helicase
phage endolysin
phage toxin-antitoxin protein MazG
hypothetical protein
phage protein
phage protein
hypothetical protein
hypothetical protein
hypothetical protein
hypothetical protein
hypothetical protein
deoxycytidylate deaminase
hypothetical protein
hypothetical protein
hypothetical protein
DNA helicase,Type III restriction enzme
phage protein
phage long tail fiber proximal subunit
phage protein
hypothetical protein
hypothetical protein
hypothetical protein
phage terminase, large subunit
phage DNA polymerase
62kDa structural protein
phage protein
phage minor capsid protein
phage protein
probable phage protein YPO2110
phage FAD/FMN-containing dehydrogenase
hypothetical protein
hypothetical protein
phage protein
phage protein
possible peptidoglycan binding protein
phage protein
phage protein
phage protein
phage tail fibers
hypothetical protein
phage tail tape measure
phage protein
phage protein

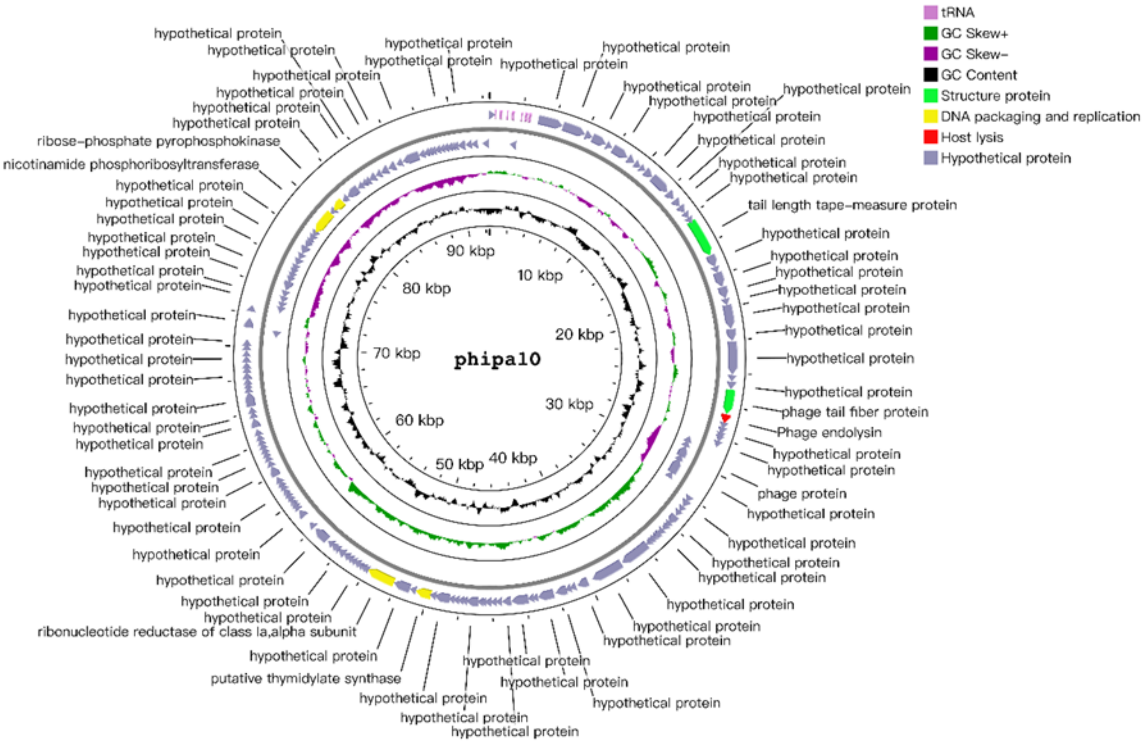
tRNA
GC Skew+
GC Skew-
GC Content
Structure protein
DNA packaging and replication
Host lysis
Hypothetical protein
phipa10
10 kbp
20 kbp
30 kbp
40 kbp
50 kbp
60 kbp
70 kbp
80 kbp
90 kbp
hypothetical protein
hypothetical protein
hypothetical protein
hypothetical protein
hypothetical protein
hypothetical protein
hypothetical protein
hypothetical protein
hypothetical protein
hypothetical protein
hypothetical protein
hypothetical protein
hypothetical protein
hypothetical protein
hypothetical protein
tail length tape-measure protein
hypothetical protein
hypothetical protein
hypothetical protein
hypothetical protein
hypothetical protein
hypothetical protein
hypothetical protein
hypothetical protein
phage tail fiber protein
Phage endolysin
hypothetical protein
hypothetical protein
phage protein
hypothetical protein
hypothetical protein
hypothetical protein
hypothetical protein
hypothetical protein
hypothetical protein
hypothetical protein
hypothetical protein
hypothetical protein
hypothetical protein
hypothetical protein
hypothetical protein
hypothetical protein
putative thymidylate synthase
hypothetical protein
ribonucleotide reductase of class Ia,alpha subunit
hypothetical protein
hypothetical protein
hypothetical protein
hypothetical protein
hypothetical protein
hypothetical protein
hypothetical protein
hypothetical protein
hypothetical protein
hypothetical protein
hypothetical protein
hypothetical protein
hypothetical protein
hypothetical protein
hypothetical protein
hypothetical protein
hypothetical protein
hypothetical protein
hypothetical protein
hypothetical protein
hypothetical protein
nicotinamide phosphoribosyltransferase
ribose-phosphate pyrophosphokinase
hypothetical protein
hypothetical protein
hypothetical protein
hypothetical protein
hypothetical protein
hypothetical protein
